# Scrutinising the Conformational Ensemble of the Intrinsically Mixed-Folded Protein Galectin-3

**DOI:** 10.3390/molecules29122768

**Published:** 2024-06-11

**Authors:** Midhun Mohan Anila, Paweł Rogowski, Bartosz Różycki

**Affiliations:** Institute of Physics, Polish Academy of Sciences, Al. Lotników 32/46, 02-668 Warsaw, Poland; midhun@ifpan.edu.pl (M.M.A.); progowski@ifpan.edu.pl (P.R.)

**Keywords:** galectin-3, conformational ensemble, fuzzy complexes, molecular dynamics simulations, Martini 3

## Abstract

Galectin-3 is a protein involved in many intra- and extra-cellular processes. It has been identified as a diagnostic or prognostic biomarker for certain types of heart disease, kidney disease and cancer. Galectin-3 comprises a carbohydrate recognition domain (CRD) and an N-terminal domain (NTD), which is unstructured and contains eight collagen-like Pro-Gly-rich tandem repeats. While the structure of the CRD has been solved using protein crystallography, current knowledge about conformations of full-length galectin-3 is limited. To fill in this knowledge gap, we performed molecular dynamics (MD) simulations of full-length galectin-3. We systematically re-scaled the solute–solvent interactions in the Martini 3 force field to obtain the best possible agreement between available data from SAXS experiments and the ensemble of conformations generated in the MD simulations. The simulation conformations were found to be very diverse, as reflected, e.g., by (i) large fluctuations in the radius of gyration, ranging from about 2 to 5 nm, and (ii) multiple transient contacts made by amino acid residues in the NTD. Consistent with evidence from NMR experiments, contacts between the CRD and NTD were observed to not involve the carbohydrate-binding site on the CRD surface. Contacts within the NTD were found to be made most frequently by aromatic residues. Formation of fuzzy complexes with unspecific stoichiometry was observed to be mediated mostly by the NTD. Taken together, we offer a detailed picture of the conformational ensemble of full-length galectin-3, which will be important for explaining the biological functions of this protein at the molecular level.

## 1. Introduction

Galectins are soluble proteins that contain at least one carbohydrate recognition domain (CRD) but no other types of folded protein domains [1,2]. They bind specifically to β-galactosides. Their molecular weights range from about 14 to 39 kDa [2,3]. In mammals, the galectin family consists of 15 members, which are involved in many biological processes, including immune responses, cell migration, autophagy and signaling [4]. Within this protein family, galectin-3 is unique in the sense that it bears a chimeric molecular architecture. Namely, it consists of a single CRD and an unstructured, non-lectin N-terminal domain (NTD) containing eight collagen-like Pro-Gly-rich tandem repeats. This unique architecture enables galectin-3 not only to interact with a plethora of ligands in a carbohydrate-dependent or -independent manner but also to form oligomers [5,6,7].

Galectin-3 is shuttled between the nucleus and cytoplasm [8]. It is also secreted to the cell surface and into extra-cellular fluids [9]. The different locations of galectin-3 contribute to its diverse functions, which include cell growth, cell adhesion, angiogenesis, apoptosis and mRNA processing [10]. Overexpression or changes in the localization of galectin-3 are commonly seen in various types of cancers. Increasing evidence indicates that galectin-3 is required for the regulation of various cell activities during cancer development, progression and metastasis [10]. Consequently, galectin-3 has been identified as a biomarker for disease diagnosis and a target for therapy [11].

Galectin-3 has been studied extensively using molecular biophysics and structural biology methods. In particular, the structure of the CRD has been solved using protein crystallography, revealing in atomic detail the carbohydrate-binding specificity of galectin-3 [12,13]. Small-angle X-ray scattering (SAXS) experiments have demonstrated that the NTD is unstructured and that the radius of gyration of the full-length galectin-3 molecule in solution is about 2.9 nm [7]. Nuclear magnetic resonance (NMR) methods have been used to study the inter- and intra-molecular interactions of galectin-3 [6,7]. However, despite this significant research progress, the current knowledge about the conformational dynamics of full-length galectin-3 is still limited. To fill in this knowledge gap, we performed molecular dynamics (MD) simulations of full-length galectin-3. We used the Martini 3 force field [14] and, following the approach of Thomasen et al. [15], re-scaled the protein–water interactions in such a way that the ensemble of conformations generated in the MD simulations was in good quantitative agreement with the data from SAXS experiments [7]. Our simulation results revealed the following picture: Full-length galectin-3 exhibits large-scale fluctuations between diverse compact and extended conformations, with the radius of gyration ranging from about 2 to 5 nm. The NTD appears to be as flexible as a Gaussian chain, and virtually any pair of amino acid residues in the NTD makes transient contacts. Many different residues in the NTD make transient contacts with the same regions on the CRD surface, which is a characteristic of fuzzy interactions, as identified in the NMR experiments [7]. Aromatic residues in the NTD make rather frequent contacts, both with the CRD and within the NTD. The convex face of the CRD comes into contact with the NTD significantly more often than the concave face of the CRD, which appears to be qualitatively consistent with enhanced chemical shifts for residues 200–220 located on the convex side of the CRD [7]. Moreover, the carbohydrate-binding site—positioned on the concave surface of the CRD—is practically never occluded by the NTD, which potentially has biological implications. Taken together, our simulation results are in good quantitative agreement with the SAXS data and qualitatively consistent with conclusions drawn from the NMR experiments, and provide a detailed account of the conformational ensemble of full-length galectin-3 under SAXS experimental conditions.

The CRD of galectin-3 has been studied to some extent using MD methods. Specifically, MD simulations have elucidated the mechanisms of oligosaccharide recognition in the CRD [16] and revealed the mode of CRD binding to ganglioside GM1 in a model lipid membrane [17]. In addition, a combination of NMR and MD has been used to explore interactions between the NTD and the CRD in full-length galectin-3 [18]. In contrast to the aforementioned studies employing all-atom MD simulations, we used the coarse-grained Martini 3 model to explore the conformational dynamics as well as the intra- and inter-molecular interactions of full-length galectin-3.

MD simulations of intrinsically disordered proteins (IDPs) are often challenging and time consuming. Firstly, IDPs typically exhibit large-scale conformational fluctuations that occur on long time scales. Therefore, the simulation time has to be sufficiently long to capture the IDP dynamics [19]. Secondly, the simulation box has to be large enough to accommodate the most extended conformations of the IDP under study; and the computational cost of explicit solvent simulations certainly increases as the simulation box is made larger. One way to significantly reduce the computational cost of MD simulations is to use coarse-grained models, where groups of atoms are represented by beads. Although coarse-grained models have a lower spatial resolution and weaker predictive power than all-atom models, they have proven very useful in diverse studies on biomacromolecules, including IDPs [15,20].

One of the most commonly used coarse-grained models for simulating biomacromolecules is currently Martini 3 [14]. We performed the MD simulations of full-length galectin-3 using Martini 3, which allowed us to study the conformational dynamics and inter-molecular interactions of this IDP on time scales up to 100 μs. The MD simulation results show, in particular, that the carbohydrate-binding site on the CRD surface barely ever makes intra-molecular contacts with the NTD and virtually no inter-molecular contacts at the protein concentration of 400 μM. These results imply that neither inter- nor intra-molecular interactions occlude the carbohydrate-binding site on the CRD surface. The MD simulations show also, consistent with experimental evidence [7,21], that interactions between galectin-3 molecules are mediated mostly by the NTDs. These results together may be important for explaining the biological functions of galectin-3 at the molecular level.

## 2. Results

**Figure 1:**
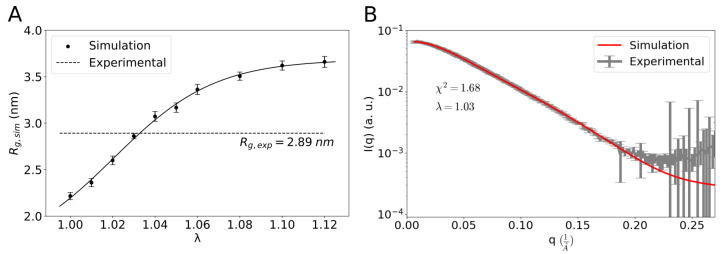
(**A**) The average radius of gyration, $R_{g,\mathrm{sim}}$, as a function of the protein–water interaction re-scaling parameter λ. The error bars indicate standard deviation calculated over a single trajectory using block averaging. The solid line is shown to guide the eye. The dashed line indicates $R_{g,\exp}=2.89$ nm, obtained from SAXS experiments [7]. (**B**) Comparison of the SAXS experimental data taken from Ref. [7] (gray points with error bars) with the scattering intensity profile obtained from the ensemble of the simulation structures with λ=1.03 (red line).

We performed MD simulations of full-length galectin-3 using the Martini 3 force field [14] and noticed that the simulation structures were, on average, significantly more compact than those observed in SAXS experiments [7]. Indeed, from the MD simulations, we obtained the average radius of gyration $R_{g,\mathrm{sim}}=2.22\pm 0.08$ nm, which was clearly smaller than $R_{g,\exp}=2.89$ nm determined from the SAXS experimental data [7]. Therefore, we followed the approach of Thomasen et al. [15] and re-scaled the protein–water interactions by a factor λ in the Martini 3 force field. Specifically, we performed a series of MD simulations of one galectin-3 molecule in a cubic box of side length L=20 nm with λ=1,1.01,…1.1,1.12. As expected, the average radius of gyration, $R_{g,\mathrm{sim}}$, was found to increase monotonically with λ (Figure 1A). At λ=1.03, we obtained $R_{g,\mathrm{sim}}=2.86\pm 0.06$ nm, consistent with the $R_{g,\exp}=2.89$ nm obtained from the SAXS experiment [7].

We quantitatively compared the SAXS experimental data with the ensemble of galectin-3 structures generated in the MD simulations with λ=1.03 (Figure 1B). Firstly, we computed scattering intensity profiles for all of the simulation structures individually. Then, we averaged the scattering intensity profiles and used the least-squares method to fit the average profile to the SAXS data published by Lin et al. [7]. We found that the ensemble-averaged scattering intensity profile was in good agreement with the SAXS data ($\chi^{2}=1.68$, Figure 1B), validating our simulation outcome.

**Figure 2:**
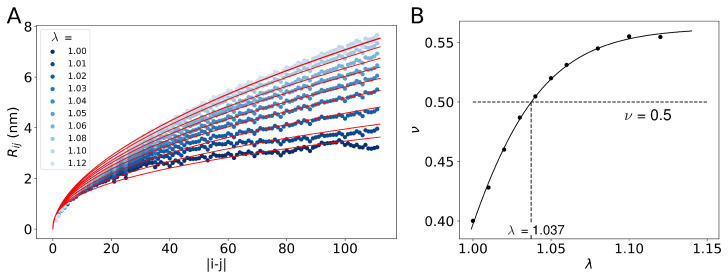
(**A**) The average inter-residue distance $R_{ij}$ as a function of sequence separation |i−j|, where *i* and *j* denote indices of residues in the NTD. The points in different hues of blue represent $R_{ij}$ values obtained from the MD simulation trajectories with different values of λ, as indicated in the inset. The solid lines in red represent fits of the function $R_{ij}\left(\nu \right)=b{\left|i-j\right|}^{\nu }$ to the simulation data, where b=0.55 nm, and ν is the Flory scaling exponent. (**B**) The scaling exponent ν as a function of parameter λ. The points in black represent the results of fitting in panel (**A**). The solid line represents a smooth fit to the points shown in black. The intersection of ν(λ) with $\nu =\frac{1}{2}$ yields the θ-point at λ=1.037.

Interestingly, the re-scaling of the protein–water interactions by just a few percent resulted in significant changes in the average radius of gyration (Figure 1A), which suggested that the spatial extent of the NTD could depend sensitively on the value of parameter λ. We set out to investigate this effect. To this end, we followed the approach of Dignon et al. [22] and computed the average inter-residue distance $R_{ij}$ as a function of sequence separation |i−j|, with *i* and *j* being indices of residues in the NTD (Figure 2A). For each of the λ-values, we fitted the resulting distances $R_{ij}$ to the function $R_{ij}\left(\nu \right)=b{\left|i-j\right|}^{\nu }$, where the Kuhn length *b* was set to 0.55 nm, as suggested for IDPs [22,23,24]. We found the Flory scaling exponent ν increased monotonically from about 0.4 to 0.55 as λ was increased from 1 to 1.1 (Figure 2B). Thus the solvent equality could be conveniently controlled by tuning the value of parameter λ. The θ-point defined by $\nu =\frac{1}{2}$ was identified at λ=1.037. Interestingly, the λ-value at which the ensemble of simulation structures was found to fit the SAXS data best, λ=1.03, was very close to the θ-point with λ=1.037, at which the NTD should effectively behave like a Gaussian chain.

**Figure 3:**
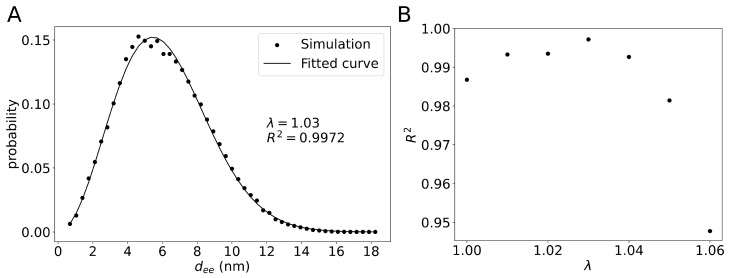
(**A**) Distribution of the end-to-end distance $d_{\mathrm{ee}}$. The points represent results obtained from the MD simulation with λ=1.03. The solid line shows the Gaussian chain end-to-end distance distribution $p\left(d_{\mathrm{ee}}\right)=4\;d_{\mathrm{ee}}^{2}\;\exp\left[-{\left(d_{\mathrm{ee}}/\xi \right)}^{2}\right]/\left(\sqrt{\pi }\;\xi^{3}\right)$ with ξ=5.44 nm. (**B**) The Pearson correlation coefficient $R^{2}$ obtained by fitting the function $p(d_{\mathrm{ee}})$ to the end-to-end distance distributions from the MD simulations with different λ-values.

We also determined distributions of the end-to-end distance $d_{\mathrm{ee}}$ for the different values of parameter λ. The end-to-end distance $d_{\mathrm{ee}}$ was defined simply as the distance between the N-terminus and the C-terminus of galectin-3. We noticed that, for λ=1.03, the end-to-end distance distribution was approximated very well by the Gaussian chain end-to-end distance distribution $p\left(d_{\mathrm{ee}}\right)=4\;d_{\mathrm{ee}}^{2}\;\exp\left[-{\left(d_{\mathrm{ee}}/\xi \right)}^{2}\right]/\left(\sqrt{\pi }\;\xi^{3}\right)$ [25] with the fitting parameter ξ=5.44 nm (Figure 3A). More generally, the end-to-end distance distributions obtained from the MD simulations could be fitted well to the function $p(d_{\mathrm{ee}})$ with the Pearson correlation coefficient $R^{2}>0.98$ as long as λ<1.06 (Figure 3B). For λ>1.06, we obtained much worse fits with $R^{2}<0.9$, indicating that conformations of the NTD were significantly different to Gaussian chain conformations.

**Figure 4:**
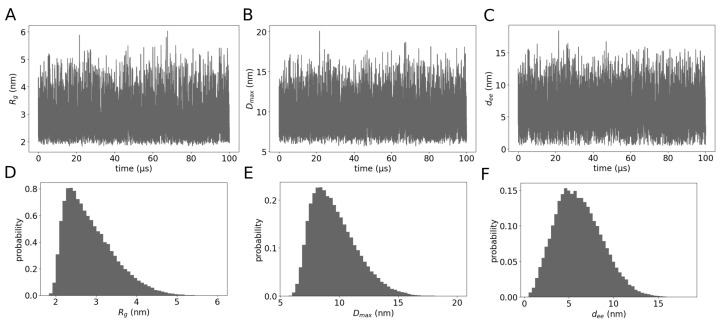
(**A**–**C**) The gyration radius $R_{g}$, the maximum diameter $D_{\max}$ and the end-to-end distance $d_{\mathrm{ee}}$ in the course of the MD simulation with λ=1.03. (**D**–**F**) Histograms of $R_{g}$, $D_{\max}$ and $d_{\mathrm{ee}}$ generated from the time series shown in panels (**A**–**C**).

We quantified the ensemble of the full-length galectin-3 structures generated in the MD simulations with λ=1.03. Firstly, we computed the gyration radius $R_{g}$, the maximum diameter $D_{\max}$ and the end-to-end distance $d_{\mathrm{ee}}$ for each of the simulation structures. The maximum diameter was defined as the largest distance between any pair of amino acid residues in the protein. We found galectin-3 undergoes large conformational fluctuations, with $R_{g}$ ranging from 2 to 6 nm (Figure 4A,D), $D_{\max}$ fluctuating in the range from about 6 to 18 nm (Figure 4B,E) and $d_{\mathrm{ee}}$ varying between 1 and 16 nm (Figure 4C,F). The values of $D_{\max}$ and $d_{\mathrm{ee}}$ were thus smaller by at least a couple of nanometers than the simulation box size *L* = 20 nm, ensuring that galectin-3 did not interact with its periodic image during the MD simulations. In addition, we found the fluctuations in $R_{g}$, $D_{\max}$ and $d_{\mathrm{ee}}$ to take place on time scales much shorter than the simulation time of 100 μs (Figure 4A–C), indicating that the simulation system was at equilibrium. We also noticed the distributions of $R_{g}$ and $D_{\max}$ to be asymmetric, exhibiting rather long tails (Figure 4A,B). The latter observation suggests that the NTD of galectin-3 undergoes spontaneous transitions between compact and extended conformations.

**Figure 5:**
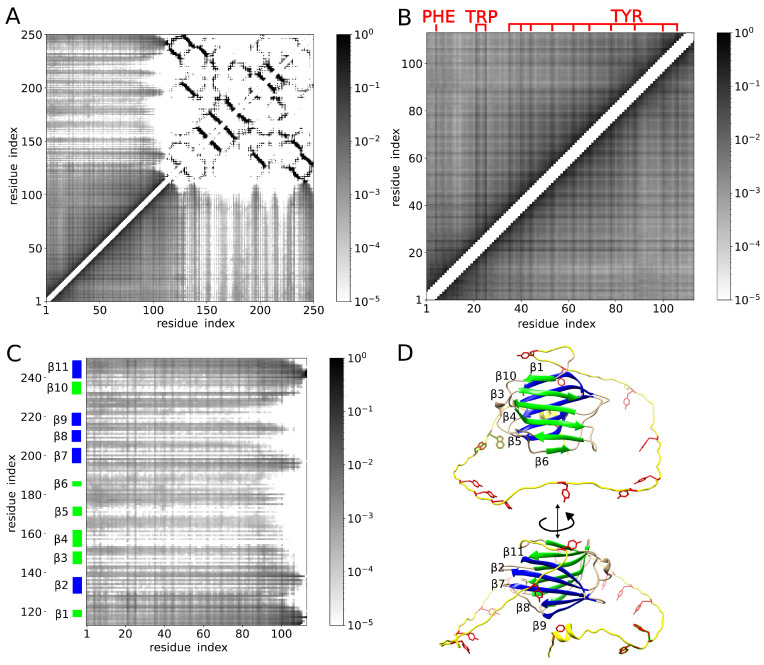
(**A**) Map of contacts between pairs of amino acid residues. The hues of gray correspond to the decimal logarithm of the contact probability. The frequent and rare contacts are marked in dark and light gray, respectively. Contacts between amino acid residues that are close in sequence (namely, contacts between residue pairs (i,i+1), (i,i+2) and (i,i+3)) are excluded from the map. (**B**) Map of contacts between amino acid residues within the NTD. The location of aromatic residues (PHE, TRP and TYR) in the amino acid sequence is highlighted in red. (**C**) Map of residue contacts between the NTD and the CRD. The β-strands within the CRD are indicated on the vertical axis. The β-strands on the concave and convex side of the CRD are marked in green and blue, respectively. (**D**) Cartoon of the galectin-3 molecule. The NTD is marked in yellow, except for the aromatic residues, which are shown in red in the stick representation. The concave face of the CRD is shown in green. It consists of β-strands $\beta_{1}$, $\beta_{10}$, $\beta_{3}$, $\beta_{4}$, $\beta_{5}$, and $\beta_{6}$. Notably, the carbohydrate-binding site is formed by $\beta_{4}$, $\beta_{5}$ and $\beta_{6}$. The convex face of the CRD is shown in blue. It consists of β-strands $\beta_{11}$, $\beta_{2}$, $\beta_{7}$, $\beta_{8}$ and $\beta_{9}$.

Next, we determined contacts between amino acid residues in each of the simulation structures and analyzed how many times each of the contacts was present in the structural ensemble. The resulting map of contacts is shown in Figure 5A, where the hues of gray correspond to the decimal logarithm of contact probability. Contacts found frequently in the structural ensemble are marked in dark gray. Rare contacts are marked in light gray. Contacts between amino acid residues that are close in sequence (i.e., contacts between residue pairs (i,i+1), (i,i+2) and (i,i+3), where index *i* labels the amino acid residues) are excluded from the map. The upper-right part of the map shows contacts within the CRD. These are mostly contacts between β-strands stabilizing the CRD structure. The lower-left part of the map corresponds to contacts within the NTD. The lower-right and upper-left parts of the map show contacts between the NTD and the CRD.

Interestingly, inspection of the contact map revealed contacts between virtually all pairs of amino acid residues in the NTD (Figure 5B), indicating a large diversity of conformations of the NTD. However, we found that the most frequent contacts within the NTD were those involving aromatic residues (Figure 5B). In addition, we found the aromatic residues in the NTD making relatively frequent contact also with many amino acid residues of the CRD (Figure 5C). However, we also noticed the NTD contacting only very rarely certain regions of the CRD (Figure 5C), including the β-strands $\beta_{4}$ and $\beta_{5}$ in particular.

**Figure 6:**
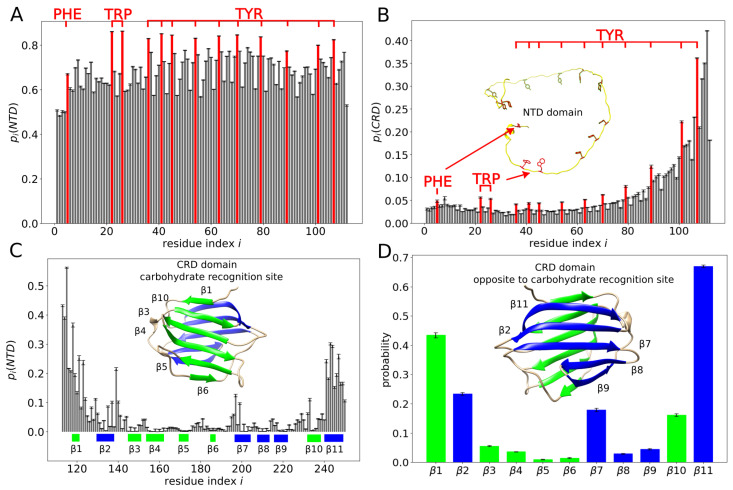
(**A**–**C**) Probability $p_{i}\left(D\right)$ of contact between a given residue with index *i* and any residue within domain *D* = NTD or CRD. The color code is as in Figure 5. (**A**) $p_{i}\left(\mathrm{NTD}\right)$ for *i* within the NTD. (**B**) $p_{i}\left(\mathrm{CRD}\right)$ for *i* within the NTD. (**C**) $p_{i}\left(\mathrm{NTD}\right)$ for *i* within the CRD. (**D**) Probability of contact between the NTD and a specific β-strand in the CRD.

To further explore these observations, we computed the probability $p_{i}\left(D\right)$ of contact between a given residue with index *i* and any residue within domain *D* = NTD or CRD. Contacts between residue pairs (i,i+1), (i,i+2) and (i,i+3) were excluded from the analysis. We found $p_{i}\left(\mathrm{NTD}\right)>0.5$ for any residue *i* in the NTD (Figure 6A). Moreover, for almost all aromatic residues in the NTD, we found $p_{i}\left(\mathrm{NTD}\right)\approx 0.8$, which was clearly larger than for other residues in the NTD (Figure 6A). The aromatic residues in the NTD were found to exhibit also larger probabilities $p_{i}\left(\mathrm{CRD}\right)$ than residues in their vicinity (Figure 6B). The residues in the CRD that we found to exhibit the largest probabilities $p_{i}\left(\mathrm{NTD}\right)$ of contact with the NTD were those within and around the β-strands $\beta_{1}$ and $\beta_{11}$ (Figure 6C,D). Most of the residues forming β-strands $\beta_{3}$, $\beta_{4}$, $\beta_{5}$, $\beta_{6}$, $\beta_{8}$ and $\beta_{9}$ were found to exhibit rather small probabilities $p_{i}\left(\mathrm{NTD}\right)$ (Figure 6C,D). In particular, we found the β-strands $\beta_{3}$ and $\beta_{4}$ making practically no contacts with the NTD (Figure 6D). This result is interesting because the carbohydrate-binding site on the CRD surface is located within the β-strands $\beta_{3}$, $\beta_{4}$ and $\beta_{5}$.

**Figure 7:**
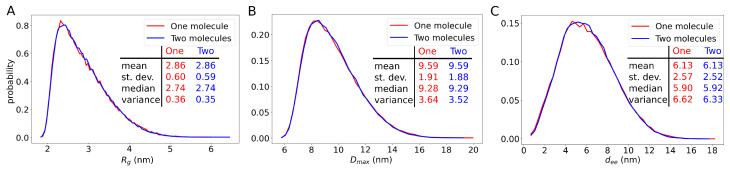
Distributions of (**A**) the radius $R_{g}$ of gyration, (**B**) the maximum diameter $D_{\max}$ and (**C**) the end-to-end distance $d_{\mathrm{ee}}$. The lines in red and blue were obtained from the MD simulations of one and two molecules of galectin-3, respectively.

**Figure 8:**
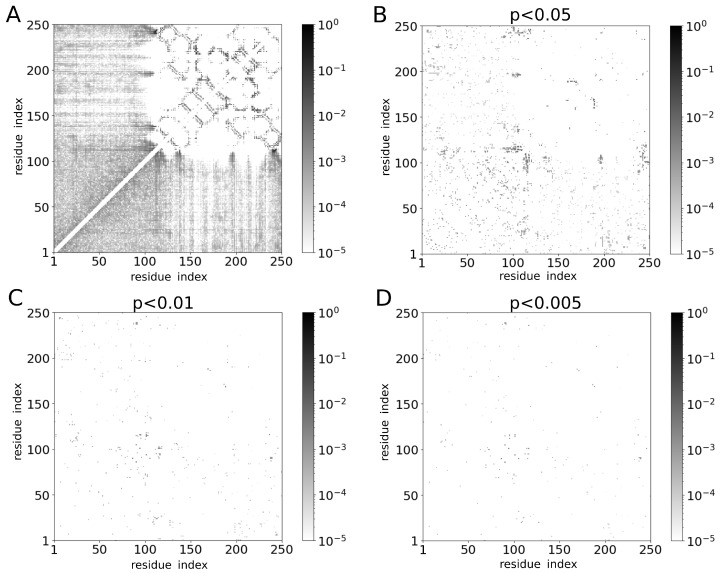
(**A**) Differences between probabilities of intra-molecular contacts observed in the MD trajectories with one and two molecules of galectin-3. The hues of gray correspond to $\log_{10}\left|p_{ij}^{\left(\mathrm{mon}\right)}-p_{ij}^{\left(\dim\right)}\right|$, where $p_{ij}^{\left(\mathrm{mon}\right)}$ and $p_{ij}^{\left(\dim\right)}$ denote the probabilities of intra-molecular contacts between residues *i* and *j* in the MD simulation with one and two molecules of galectin-3, respectively. (**B**–**D**) Analogous to panel A but for contacts with the ANOVA test *p*-values of (**B**) 0.05, (**C**) 0.01 and (**D**) 0.005.

We next performed MD simulations of two molecules of galectin-3 in a cubic box of side length L=20 nm using λ=1.03. The protein concentration in these simulations was 0.4 mM, as in the NMR experiments reported by Lin et al. [7]. We determined the gyration radius $R_{g}$ (Figure 7A), the maximum diameter $D_{\max}$ (Figure 7B) and the end-to-end distance $d_{\mathrm{ee}}$ (Figure 7C) for each of the galectin-3 structures generated in these simulations. Unexpectedly, we found that distributions of these quantities were practically identical to the corresponding distributions obtained from the MD simulations of a single molecule of galectin-3 (Figure 7), suggesting that the conformations of galectin-3 were unaffected by inter-molecular interactions at this protein concentration. To investigate this observation in more detail, we analyzed intra-molecular contacts. Specifically, we computed the probabilities $p_{ij}^{\left(\mathrm{mon}\right)}$ and $p_{ij}^{\left(\dim\right)}$ of the intra-molecular contacts between residues *i* and *j* in the MD trajectory with one and two molecules of galectin-3, respectively, and plotted a map of $|p_{ij}^{\left(\mathrm{mon}\right)}-p_{ij}^{\left(\dim\right)}|$ (Figure 8). Although differences between $p_{ij}^{\left(\mathrm{mon}\right)}$ and $p_{ij}^{\left(\dim\right)}$ were noticeable for many residue pairs (Figure 8A), the ANOVA test showed that the great majority of these differences were statistically insignificant (Figure 8B–D). We thus concluded that the intra-molecular contacts and conformations of galectin-3 were almost unaffected by inter-molecular interactions at the protein concentration of 0.4 mM (Figure S1).

**Figure 9:**
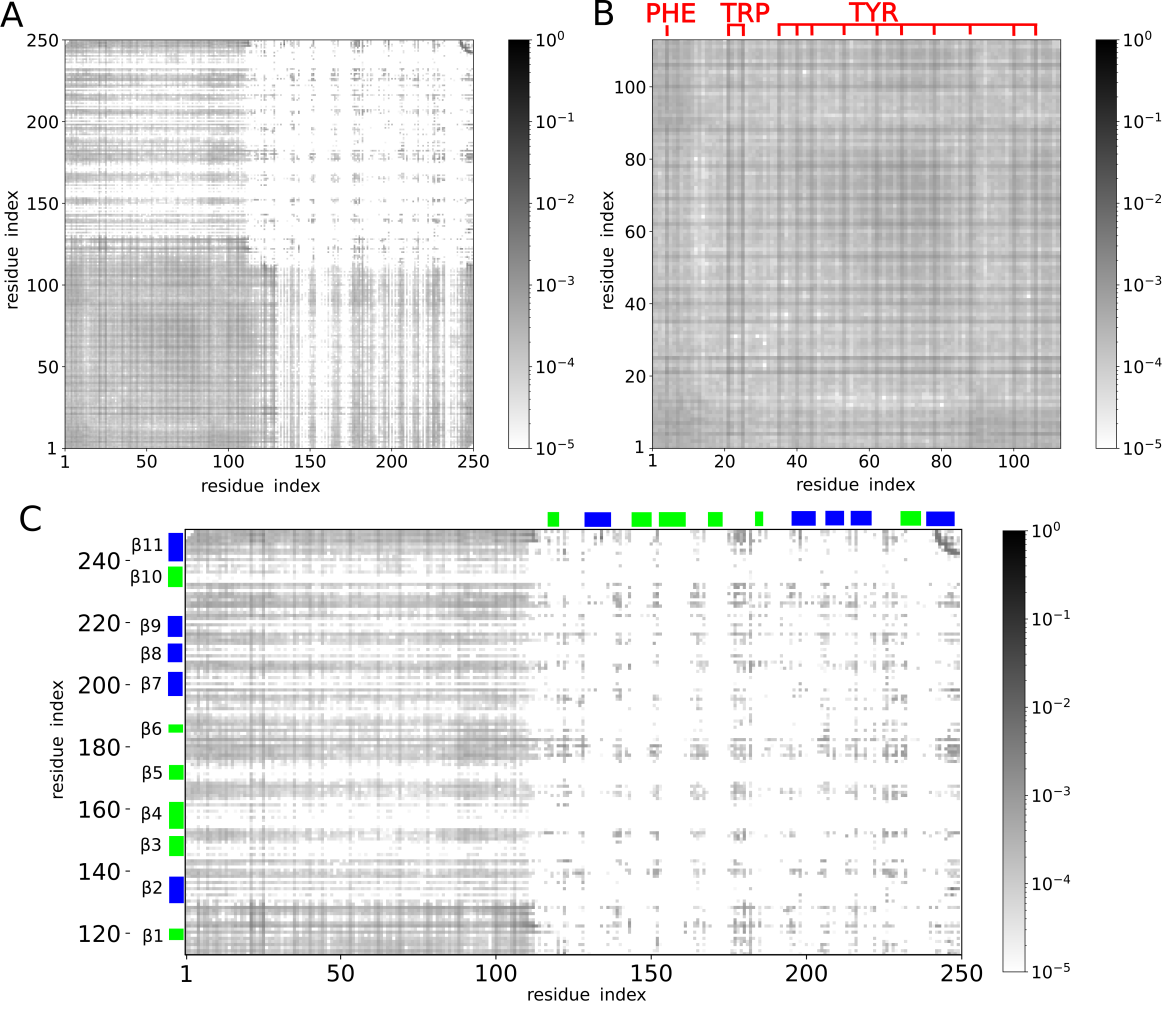
(**A**) Map of contacts between two molecules of galectin-3 at the protein concentration of 0.4 mM. The hues of gray correspond to the decimal logarithm of the contact probability. (**B**) Map of inter-molecular contacts between the NTDs. The location of aromatic residues in the amino acid sequence is highlighted in red. (**C**) Map of inter-molecular contacts formed by amino acid residues in the CRD. The β-strands on the concave and convex side of the CRD are marked in green and blue, respectively.

We analyzed the maps shown in Figure 8C,D (corresponding to the ANOVA test *p*-values of 0.01 and 0.005, respectively) and selected all contacts with $|p_{ij}^{\left(\mathrm{mon}\right)}-p_{ij}^{\left(\dim\right)}|>0.001$. Interestingly, we found that the great majority of these contacts were formed by aromatic residues and prolines (and, to a much smaller extent, also by glycines, serines and alanines) in the NTD and at the NTD–CRD interface (i.e., around residues 110 to 120). These amino acid residues (especially the aromatic ones) were also found to be involved in inter-molecular contacts (Figure 9).

We next analyzed inter-molecular contacts, i.e., contacts between the two molecules of galectin-3 (Figure 9A). We found that contacts between the NTDs were rather unspecific and fuzzy, although formed relatively often by aromatic residues (Figure 9B). The aromatic residues in the NTD were also noticed to make inter-molecular contacts with many residues on the CRD surface (Figure 9C). The amino acid residues in the CRD that were identified as making inter-molecular contacts were mostly those positioned within the loops and turns between the β-strands as well as within the β-strands $\beta_{1}$ and $\beta_{11}$ (Figure 9C).

**Figure 10:**
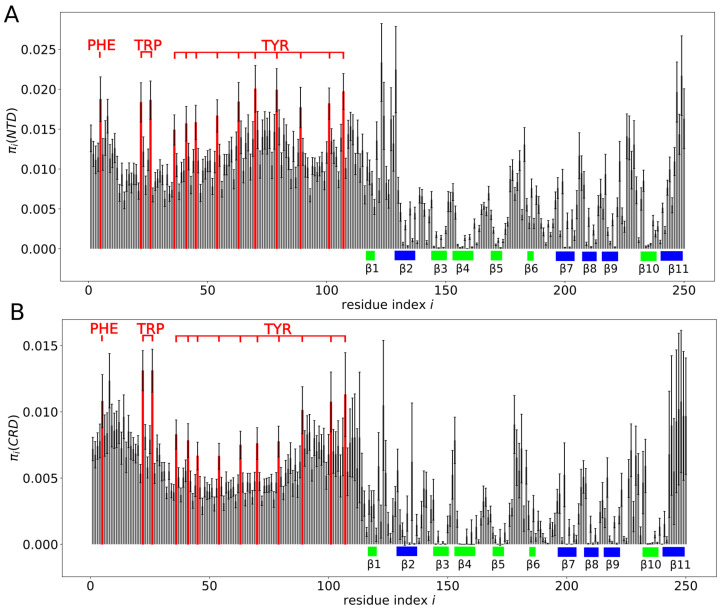
Probabilities of contact between a given residue in one molecule of galectin-3 and a given domain (**A**) NTD or (**B**) CRD of the other molecule of galectin-3. The location of aromatic residues in the NTD is highlighted in red. The β-strands on the concave and convex face of the CRD are marked in green and blue, respectively.

We also determined the probability $\pi_{i}\left(D\right)$ of contact between a given residue with index *i* in one molecule of galectin-3 and a given domain *D* = NTD or CRD of the other molecule of galectin-3 (Figure 10). We found $\pi_{i}\left(\mathrm{NTD}\right)\approx 0.01$ and $\pi_{i}\left(\mathrm{CRD}\right)\approx 0.005$ for almost any amino acid residue *i* within the NTD, as is characteristic for fuzzy inter-molecular interactions. In contrast, for amino acid residues within the CRD, both $\pi_{i}\left(\mathrm{NTD}\right)$ and $\pi_{i}\left(\mathrm{CRD}\right)$ were found to depend sensitively on the residue index *i*. In particular, we observed a vanishing $\pi_{i}\left(\mathrm{NTD}\right)$ and $\pi_{i}\left(\mathrm{CRD}\right)$ for most of residues within the β-strands $\beta_{3}$, $\beta_{4}$, $\beta_{5}$, $\beta_{6}$ and $\beta_{10}$, i.e., on the concave face of the CRD.

**Figure 11:**
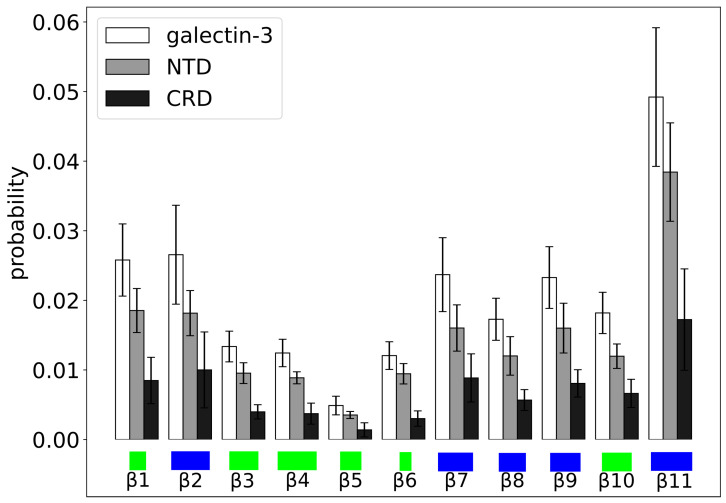
Probability of contact between a given β-strand in the CRD of one molecule of galectin-3 and the NTD (bars in gray) or the CRD (bars in black) of the other molecule of galectin-3. The β-strands on the concave and convex side of the CRD are marked in green and blue, respectively.

We also computed the probability of contact between a given β-strand ($\beta_{1},\ldots ,\beta_{11}$) in the CRD of one molecule of galectin-3 and a given domain (NTD or CRD) of the other molecule of galectin-3 (Figure 11). We found $\beta_{11}$ making inter-molecular contacts most frequently, with both the NTD and the CRD. We identified $\beta_{3}$, $\beta_{4}$, $\beta_{5}$ and $\beta_{6}$ (forming the concave face of the CRD) as the β-strands to be making inter-molecular contacts most rarely with both the NTD and the CRD. Interestingly, we found each of the β-strands making inter-molecular contacts more often with the NTD than with the CRD, indicating that the NTD–CRD contacts occurred in the MD trajectory more often than the CRD–CRD contacts. To further quantify this observation, we computed the probability $\pi (D_{1},D_{2})$ of contact between domain $D_{1}$ = NTD or CRD of one molecule of galectin-3 and domain $D_{2}$ = NTD or CRD of the other molecule of galectin-3. We obtained π(NTD,NTD)=0.14±0.03, π(NTD,CRD)=0.11±0.03 and π(CRD,CRD)=0.07±0.02, implying that interactions between galectin-3 molecules in the MD trajectory were mediated mostly by the NTDs, consistent with evidence from NMR experiments [7].

In a case of insufficient sampling in the MD simulations, inter-molecular contact probabilities (Figure 9, Figure 10 and Figure 11) may be biased by the initial placement of the galectin-3 molecules in the simulation box. To rule out this possibility, we analyzed the relative positions and orientations of the two proteins in the MD trajectory (Figure S2). We found that the distance between the centers of the two CRDs fluctuated rapidly between about 3 and 17 nm on time scales much shorter than the simulation time of 100 μs (Figure S2A). Also the relative orientation of the two CRDs was found to change quickly between 0 and 180° on time scales much shorter than 100 μs (Figure S2B). We thus concluded that 100 μs of the coarse-grained dynamics was sufficient to sample all possible relative positions and orientations of the two molecules of galectin-3 in the simulation box.

**Figure 12:**
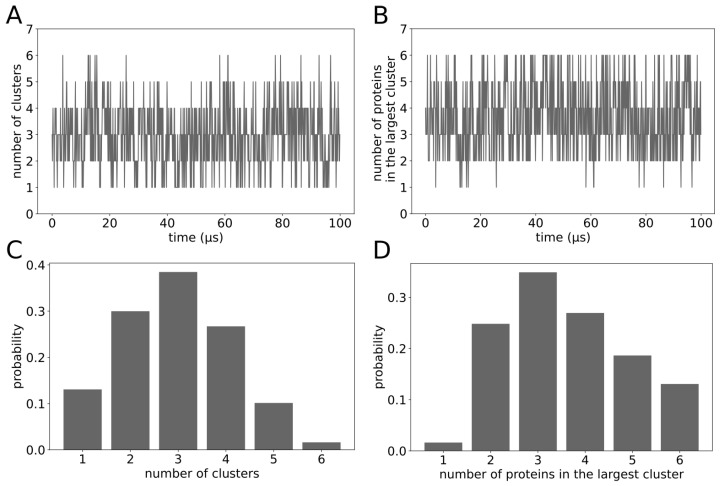
(**A**) Number of clusters and (**B**) number of proteins forming the largest cluster as a function of time. (**C**,**D**) Corresponding histograms of the number of clusters and the number of proteins forming the largest clusters. Results obtained from the MD simulations of six molecules of galectin-3 in a cubic box of side length L=20 nm with λ=1.03.

To study galectin-3 oligomerization [26], we performed MD simulations of six molecules of galectin-3 in a cubic box of side length L=20 nm with λ=1.03 and determined clusters of the protein molecules. Two molecules of galectin-3 were assigned to the same cluster if they were identified as making at least one inter-molecular contact. We determined the number of clusters (Figure 12A) and the number of molecules forming the largest cluster (Figure 12B) as a function of time. Interestingly, we found both of these numbers to fluctuate between 1 and 6, indicating that galectin-3 could form fuzzy complexes [7]. We found the most likely number of clusters to be three (Figure 12C) and the largest cluster to comprise most typically three molecules of galectin-3 (Figure 12D). However, the probability of all six molecules of galectin-3 forming one cluster was found to be over $\frac{1}{8}$ (Figure 12C,D).

## 3. Methods

### 3.1. Molecular Dynamics Simulations

A structural model of full-length galectin-3 was built based on (i) the crystal structure of the CRD deposited in the Protein Data Bank (PDB) with the entry code of 2NMO [13] and (ii) atomic coordinates of the NTD generated using the Molefacture Plugin in VMD [27]. The structural model of full-length galectin-3 was converted to the Martini 3 coarse-grained representation using Martinize2. Secondary structure elements within the CRD were assigned using DSSP [28] in Martinize2. The NTD was assigned as a random coil. An elastic network was imposed on the CRD to preserve the domain structure in the course of the MD simulations. Specifically, Martinize2 was called with flags -elastic -ef 700.0 -el 0.5 -eu 0.9 -ea 0 -ep 0 -scfix. In this way, harmonic restraints between backbone beads between the lower and upper distance cut-offs of 0.5 and 0.9 nm, respectively, were imposed. The spring constant of the harmonic restraints was set to 700 kJ mol^−1^ nm^−2^. The harmonic restraints within the NTD, as well as those between the NTD and CRD, were subsequently removed from the topology file using a Python-3 script developed by Thomasen et al. [15]. In addition, by applying the flag -scfix in Martinize2, extra torsional potentials between backbone beads and side chain beads were added [29]. These potentials were included in the force field to prevent unphysical flipping of side chains in β-strands of the CRD.

The galectin-3 coarse-grained model was placed in a cubic box of side length L=20 nm using the Gromacs editconf tool. Then, the protein was solvated using Insane [30] with sodium and chloride ions added to neutralize the simulation system and to reach the salt concentration of 100 mM. Consequently, there were 489 sodium ions and 491 chloride ions in the simulation box. The energy of the system was minimized in 200 steps of the steepest descent algorithm implemented in Gromacs. The force field parameters describing interactions between the protein and water beads were re-scaled by a factor λ using a Python script provided by Thomasen et al. [15]. The values of parameter λ were set to 1,1.01,…1.1,1.12. Then, for each of the λ-values, an equilibration simulation of 10 ns was performed with the integration time step of 2 fs to allow for slow relaxation of the system.

The MD simulations were performed using Gromacs version 2021.5. Production runs were performed with the integration time step of 20 fs. Nonbonded interactions were treated with the Verlet cut-off scheme. The cut-off for van der Waals interactions was set to 1.1 nm. Coulomb interactions were treated using the reaction field method with a cut-off of 1.1 nm and dielectric constant of 15. The temperature and pressure were kept constant at *T* = 300 K and *p* = 1 bar, respectively, using the velocity-rescaling thermostat and the Parrinello–Rahman barostat. System configurations were saved at regular intervals of 1 ns. For most of the λ-values, the simulation trajectories were 20 μs long. However, for λ=1.03, the total simulation time was 100 μs to obtain sufficient statistics for detailed analyses of amino acid residue contacts; see Section 3.2.

MD simulations of two and six molecules of galectin-3 in a cubic box of side length L=20 nm and with λ=1.03 were set up and carried out analogously. The flag -merge was used in Martinize2. The galectin-3 molecules were placed in the simulation box in random orientations using Packmol [31] with a tolerance of 0.4 nm and a maximum of 20 iterations. The protein concentration was 0.42 and 1.25 mM, respectively, in the simulations of two and six molecules of galectin-3. At each of the two concentrations of galectin-3, the MD time was 100 μs.

### 3.2. Analysis of Molecular Dynamics Trajectories

Periodic boundary conditions were handled using the Gromacs tool *trjconv* with the flags -pbc whole -center. However, to analyze inter-molecular contacts in the MD trajectories with two and six molecules of galectin-3, we used the Python script *mdvwhole* [32] with the flags -sel ’name BB SC1 SC2 SC3 SC4 SC5’ -res -0.7 instead.

The radius of gyration ($R_{g}$), the maximum diameter ($D_{\max}$) and the end-to-end distance ($d_{\mathrm{ee}}$) of galectin-3 were computed on the basis of the Cartesian coordinates of protein beads (BB, SC1, SC2, SC3, SC4 and SC5) using Python with the *MDTraj* library [33]. For a given conformation of galectin-3, $D_{\max}$ was computed as the largest of the distances between all pairs of protein beads, whereas $d_{\mathrm{ee}}$ was taken as the distance between the backbone beads of the amino acid residues at the N- and the C-terminus. Histograms of $R_{g}$, $D_{\max}$ and $d_{\mathrm{ee}}$ were determined using Python with the *NumPy* library [34]. The average value of $R_{g}$ was determined separately for each of the trajectories with specific λ-values. The statistical error of the average $R_{g}$ was estimated using block error analysis [35].

The average inter-residue distance $R_{ij}$ as a function of sequence separation |i−j| was determined from the Cartesian coordinates of the protein beads in the following way: For a given conformation of galectin-3, the distance $d_{ij}$ between two amino acid residues with indices *i* and *j* was taken as the smallest of all the distances between the beads forming residue *i* and the beads forming residue *j*. The distances $d_{ij}$ were computed using the function *compute_contacts* from the Python library *MDTraj*. The average inter-residue distance $R_{ij}$ was then obtained by averaging $d_{ij}$ over all conformations sampled in the MD simulation with a given λ-value and over all residue pairs *i* and *j* with a given sequence separation |i−j|.

For SAXS analysis, every 5th frame was extracted from the MD trajectory of one molecule of galectin-3 with λ=1.03. The resulting N=20,000 conformations of galectin-3 (recorded evenly every 5 ns in the MD trajectory) were converted from the coarse-grained Martini representation to the all-atom representation using the Backward algorithm with default parameters [36]. The Python script backward.py was used to conduct the actual backmapping. The bash wrapper initral-v5.sh was used to perform the energy minimization and position-restraint MD simulations using Gromacs with the CHARMM36m force field [37].

The resulting N=20,000 structures of galectin-3 in the all-atom representation were used as input for SAXS analysis. Namely, the scattering intensity profile was computed for each of the structures individually using Pepsi-SAXS [38]. The ensemble-averaged scattering intensity profile was computed as

$$I_{\mathrm{sim}}\left(q\right)=\frac{1}{N}\sum_{k=1}^{N}I_{k}\left(q\right)$$

where k=1,…N labels the galectin-3 structures in the ensemble, $I_{k}\left(q\right)$ is the scattering intensity profile of the *k*th structure and *q* is the X-ray momentum transfer (i.e., q=4πsin(θ/2)/λ, where θ and λ denote the scattering angle and the X-ray wavelength, respectively). The discrepancy between the SAXS experimental data, $I_{\exp}\left(q\right)$, and the ensemble-averaged scattering intensity profile, $I_{\mathrm{sim}}\left(q\right)$, was quantified by

$$\chi^{2}\left(a,b\right)=\frac{1}{n}\sum_{i=1}^{n}{\left(I_{\exp}\left(q_{i}\right)-a\;I_{\mathrm{sim}}\left(q_{i}\right)-b\right)}^{2}/\sigma_{\exp}^{2}\left(q_{i}\right)$$

where n=221 is the number of points in the experimental dataset, and σ(q) denotes the statistical error of the SAXS intensity $I_{\exp}\left(q\right)$. The scale parameter *a* and the offset *b* were fitted to minimize $\chi^{2}\left(a,b\right)$ using the Python library *SciPy* [39]. The calculations were performed with different contrasts of the hydration shell electron density δρ ranging from 4% to 10% of the bulk water electron density ρ=334 e/nm^3^. The best fit with $\chi^{2}=1.68$ was obtained for δρ=0.075ρ≈25 e/nm^3^.

Contact maps were determined using the *ContactTrajectory* function from the Python library *Contact Map Explorer 0.7.0* [40]. A pair of amino acid residues was considered to be in contact if the distance between any bead in one of the residues and any bead in the other residue was smaller than 0.7 nm. Contacts between residue pairs (i,i+1), (i,i+2) and (i,i+3) were excluded from the analysis. Consequently, the *ContactTrajectory* function was called with parameters cutoff = 0.7 and n_neighbors_ignored = 3. The same criteria were used to determine intra- and inter-molecular contacts.

The probability of contact between two residues was defined as the number of frames in which the two residues were in contact divided by the total number of frames in the MD trajectory, which was $10^{5}$ for the simulations with λ=1.03. All analyses involving the probability of contact occurrence were performed using the *ContactFrequency* function from the Python library *Contact Map Explorer 0.7.0* with parameters cutoff = 0.7 and n_neighbors_ignored = 3. The statistical error of the probability of contact occurrence was estimated using block error analysis [35].

To compute the probability of contact between a given residue and a specific domain, the NTD was assumed to comprise amino acid residues with indices from 1 to 112. Consequently, the boundaries of the CRD were taken to be amino acid residues with indices 113 and 250. To determine the probability of contact formed by a specific β-strand in the CRD, the β-strands were taken to comprise amino acid residues with the following indices: from 118 to 121 ($\beta_{1}$), from 130 to 138 ($\beta_{2}$), from 145 to 151 ($\beta_{3}$), from 154 to 162 ($\beta_{4}$), from 170 to 174 ($\beta_{5}$), from 185 to 187 ($\beta_{6}$), from 197 to 204 ($\beta_{7}$), from 208 to 213 ($\beta_{8}$), from 216 to 222 ($\beta_{9}$), from 233 to 238 ($\beta_{10}$) and from 240 to 249 ($\beta_{11}$).

To identify oligomers formed in the MD simulation with six molecules of galectin-3 in a cubic box of side length L=20 nm with λ=1.03, agglomerative clustering was applied to each of the configurations obtained from the MD simulation [41]. At the beginning of the clustering procedure, each of the galectin-3 molecules was taken as a separate cluster. Then, clusters were merged successively. Two clusters were merged if any molecule from one cluster was identified to make at least one contact with any molecule from the other cluster. The clustering procedure was stopped when no more clusters could be merged. This clustering procedure was implemented in Python and applied successively to each of the molecular coarse-grained configurations recorded in the MD simulation.

## 4. Discussion

SAXS is a useful and widely used method to characterize macromolecules in solution [42,43]. Standard analysis of SAXS data of a globular protein yields the values of $R_{g}$ and $D_{\max}$, as well as the so-called molecular envelope that represents the overall shape of the protein [44]. However, SAXS analysis of proteins containing intrinsically disordered regions requires different approaches based on generating an ensemble of protein conformations [45,46,47,48]. In particular, a combination of SAXS experiments and coarse-grained molecular simulations has proven very useful in delineating conformations of proteins comprising both folded domains and intrinsically disordered regions [49,50,51,52,53,54,55,56]. Here, we followed this approach and used coarse-grained MD simulations together with available SAXS data to explore the conformational ensemble of full-length galectin-3.

Galectin-3 has been reported to be involved in numerous intra- and extra-cellular processes [10]. In particular, galectin-3 has been demonstrated to drive glycosphingolipid-dependent biogenesis of clathrin-independent carriers [57]. Although galectin-3 oligomerization has been implicated in this process, the molecular mechanisms underlying the endocytic pit formation remain elusive. However, the discovery that galectin-3 forms biomolecular condensates sheds new light on how this protein can perform its biological functions at the cell membrane [21]. Recently, we devised a mesoscopic model with dissipative particle dynamics (DPD) to simulate biomolecular condensates of galectin-3 and their interactions with lipid membranes [41]. The DPD simulations reveal the mesoscopic mechanisms by which the biomolecular condensates can sense and generate membrane curvature but do not provide insights into molecular details of these mechanisms. In the current study, in contrast, we used MD simulations with the Martini 3 force field to explore the conformations of full-length galectin-3 in solution. We slightly altered the magnitude of protein–water interactions in the the force field to obtain an ensemble of galectin-3 conformations in good quantitative agreement with available data from SAXS experiments [7] (Figure 1). We quantified the conformational ensemble in terms of protein size, flexibility and intra-molecular contacts (Figure 4, Figure 5 and Figure 6). Interestingly, we found that the conformational ensemble at infinite dilution—as obtained from the MD simulations of a single molecule of galectin-3—was barely distinguishable from the conformational ensemble at the protein concentration of 400 μM, i.e., obtained from the MD simulations of two molecules of galectin-3 in the cubic box of side length L=20 nm (Figure 7 and Figure 8) This result indicates that the protein concentration range between 40 and 400 μM used in the NMR experiments [7] is appropriate for telling apart the intra- and inter-molecular interactions.

Dignon et al. have identified a strong correlation between the critical temperature and the θ-point temperature of IDPs [22]. Our simulation results show that the galectin-3 SAXS experimental conditions are very close to the θ-point conditions (Figure 2B and Figure 3A) and that galectin-3 molecules associated only transiently form to diverse oligomers at elevated concentrations (Figure 12), indicating together that galectin-3 does not aggregate or condensate at these conditions. As a matter of fact, galectin-3 has been shown to undergo liquid–liquid phase separation only at relatively high salt concentrations [21]. Future MD studies may help to elucidate this phenomenon and delineate the structure of galectin-3 condensates.

## 5. Conclusions

Our detailed analysis of the MD trajectory with one molecule of galectin-3 shows that (i) full-length galectin-3 exhibits large-scale fluctuations between diverse compact and extended conformations (Figure 4 and Figure 5); (ii) the NTD is practically as flexible as a Gaussian chain (Figure 3A), and virtually any pair of amino acid residues in the NTD makes transient contacts (Figure 5B); (iii) aromatic residues in the NTD make the most frequent intra-molecular contacts—both with the CRD and within the NTD (Figure 5); (iv) many different residues in the NTD make transient contacts with single regions on the CRD surface (Figure 5C), which is a characteristic of fuzzy interactions and has been identified in NMR experiments [7]; and (v) the convex face of the CRD comes into contact with the NTD significantly more often than the concave face of the CRD (Figure 5 and Figure 6), which appears to be consistent with NMR chemical shifts for residues 200–220 located on the convex side of the CRD [7]. On the one hand, such a detailed picture of the conformational ensemble of full-length galectin-3 cannot be at present obtained from experiments alone. On the other hand, our simulation results are not only in good quantitative agreement with the data from SAXS experiments ($\chi^{2}=1.68$, Figure 1B), but also consistent with evidence from the NMR experiments [7].

Our analysis of the MD trajectories with two and six molecules of galectin-3 shows additionally that the β-strands $\beta_{4}$, $\beta_{5}$ and $\beta_{6}$ (forming the carbohydrate-binding site on the CRD surface) barely ever make intra-molecular contacts with the NTD (Figure 6) and virtually no inter-molecular contacts at the protein concentration of 400 μM (Figure 10 and Figure 11). These results mean that neither inter- or intra-molecular interactions occlude the carbohydrate-binding site on the CRD surface, which potentially has biological implications. In addition, consistent with experimental evidence [7], the MD simulations show that interactions between galectin-3 molecules are mediated mostly by the NTDs (Figure 9, Figure 10 and Figure 11).

## Data Availability

The original contributions presented in the study are included in the article and Supplementary Material. Further inquiries can be directed to the corresponding author.

## Supplementary Materials

The following supporting information can be downloaded at https://www.mdpi.com/article/10.3390/molecules29122768/s1: Figure S1: Data supporting Figure 8, Figure S2: Data supporting Figure 9, Figure S3: Snapshots from subsequent quarters of the MD trajectory of one molecule of galectin-3 with λ = 1.03, Figure S4: Snapshots from subsequent quarters of the MD trajectory of two molecules of galectin-3, Figure S5: Snapshots from subsequent quarters of the MD trajectory of six molecules of galectin-3.
