# Supplementary material for: Scrutinising the Conformational Ensemble of the Intrinsically Mixed-Folded Protein Galectin-3
Source: Molecules. 2024 Jun 11;29(12):2768. doi: 10.3390/molecules29122768 (PMC11207097; doi:10.3390/molecules29122768)
Supplement: Supplementary file 1 [file molecules-29-02768-s001.zip › molecules-3007684-supplementary.pdf]

Scrutinising the Conformational Ensemble of the Intrinsically Mixed-Folded Protein Galectin-3: Supporting Information

Midhun Mohan Anila, Paweł Rogowski and Bartosz Różycki
Institute of Physics, Polish Academy of Sciences
Al. Lotników 32/46, 02-668 Warsaw, Poland

1 Supporting Figures

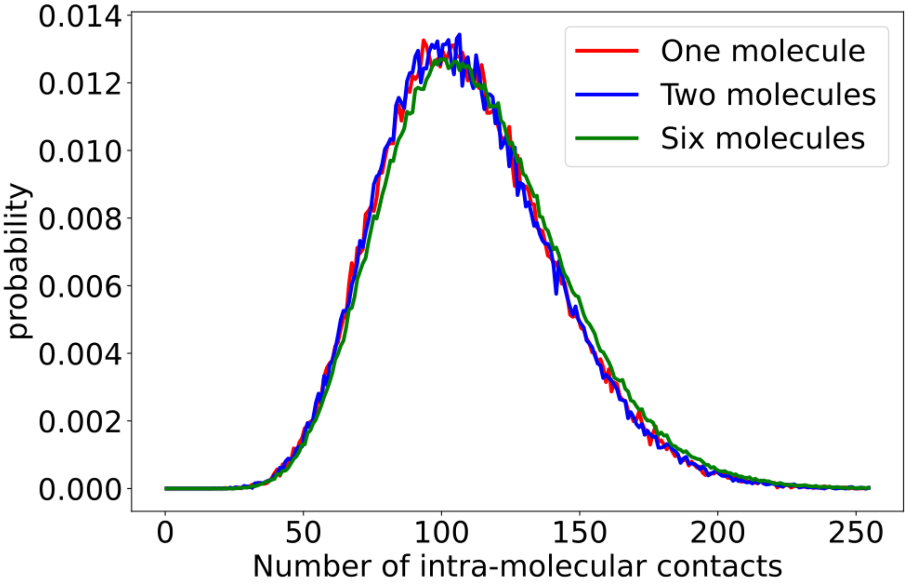

Figure S1: Data supporting Figure 8. Distributions of the number of intra-molecular contacts as obtained from the simulations of one (red), two (blue) and six (green) molecules of galectin-3.

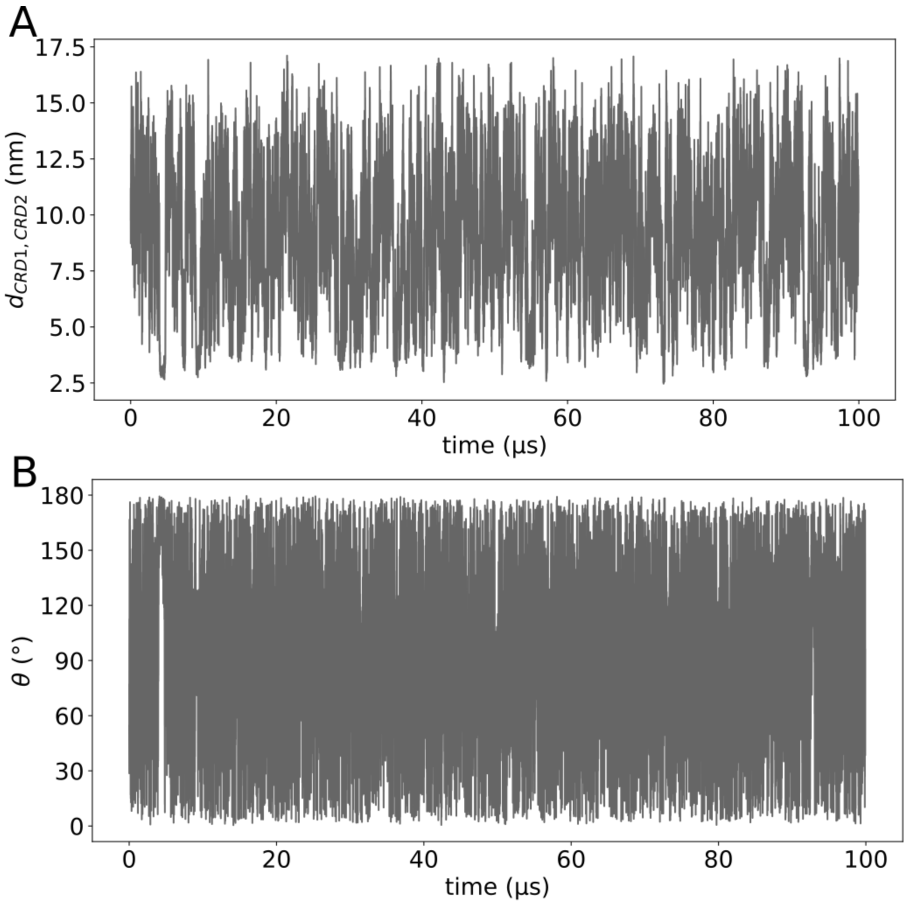

Figure S2: Data supporting Figures 9. (A) Distance $d_{\mathrm{CDR1,CRD2}}$ between the centers of the two CRDs as a function of time. (B) Angle θ of the relative orientation of the two CRDs as a function of time. The orientation of a given CRD relative to the simulation box frame is defined here by a vector joining the backbone beads of LYS226 and ASP241. The relative orientation of the two CRDs is given by the angle θ between the two vectors of the CRD orientation.

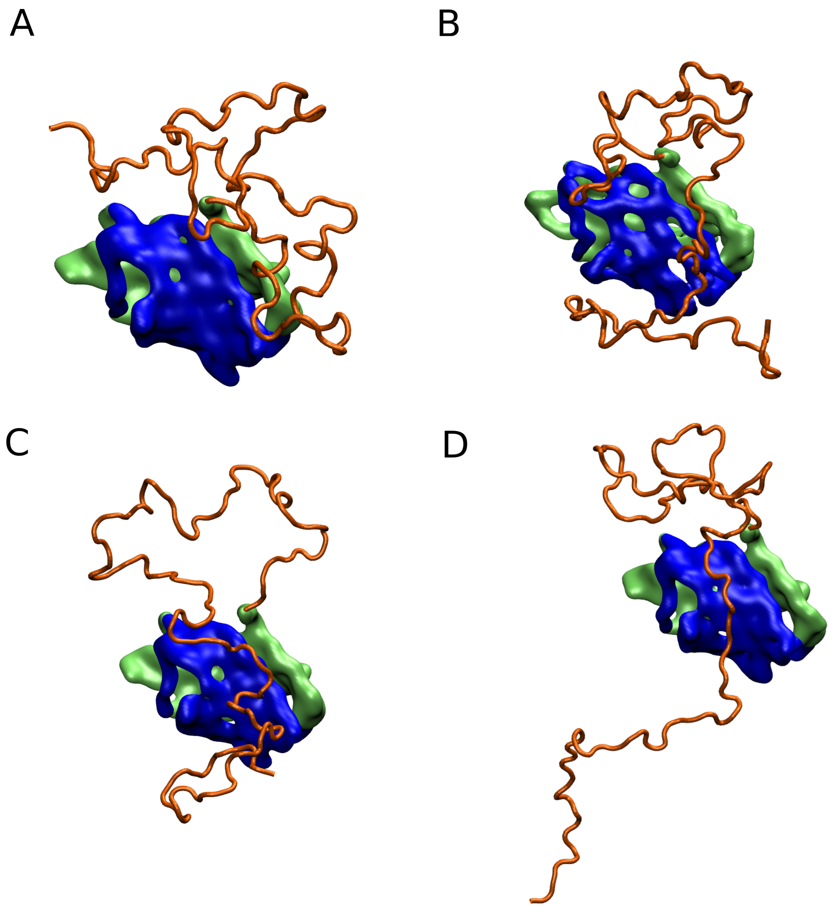

Figure S3: Snapshots from subsequent quarters of the MD trajectory of one molecule of galectin-3 with $\lambda = 1.03$. The disordered NTD is shown in a cartoon representation in orange. The folded CRD is shown in a surface representation with the convex and concave surface marked in blue and green, respectively. The snapshots are selected to illustrate diverse modes of NTD-CRD intra-molecular interactions.

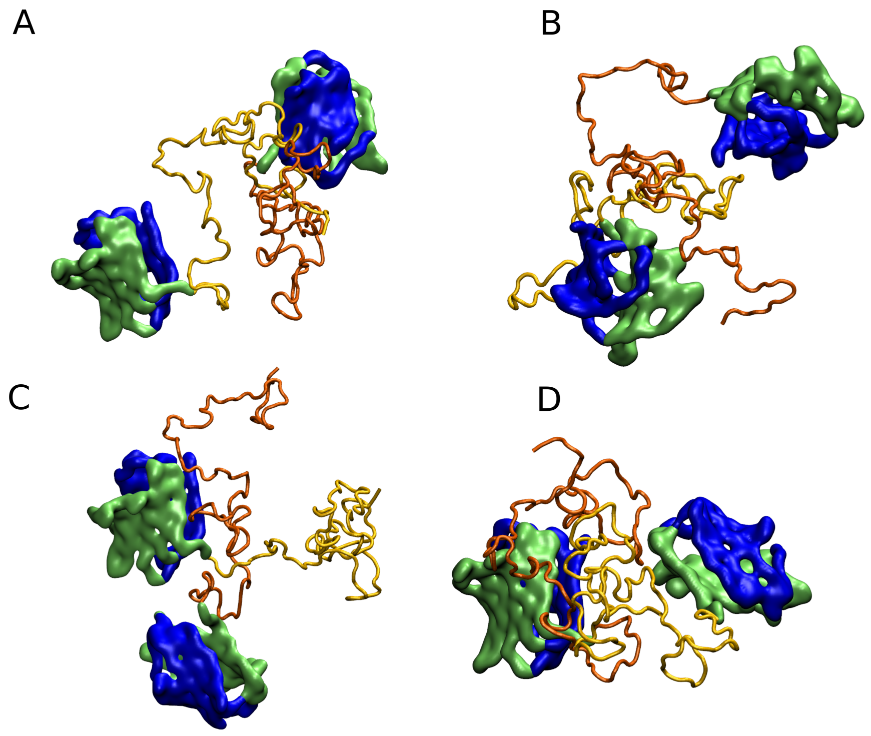

Figure S4: Snapshots from subsequent quarters of the MD trajectory of two molecules of galectin-3. The color code is an in Fig. S3. The snapshots are selected to illustrate diverse inter-molecular interactions.

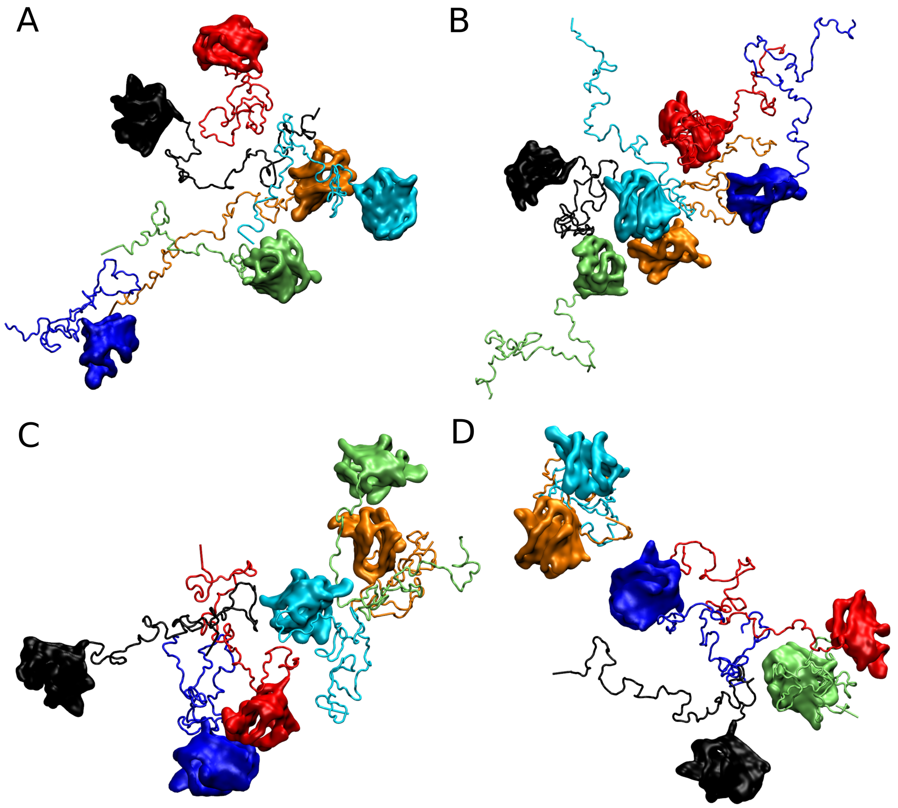

Figure S5: Snapshots from subsequent quarters of the MD trajectory of six molecules of galectin-3. Each of the galectin-3 molecules is shown in a different color. The NTDs are shown in a cartoon representation. The CRDs are shown in a surface representation. The snapshots are selected to illustrate diverse inter-molecular interactions in clusters where all of the galectin-3 molecules are in contact.
